# Divergent and stabilizing selection shape the phenotypic space of *Arabidopsis thaliana*

**DOI:** 10.1371/journal.pbio.3003536

**Published:** 2025-12-01

**Authors:** Maria Stefania Przybylska, Cyrille Violle, Denis Vile, J. F. Scheepens, Denis Cornet, Gregory Beurier, Lauriane Rouan, Aurélien Estarague, Elena Kazakou, Lucie Mahaut, François Munoz, Detlef Weigel, Moises Exposito-Alonso, Oliver Bossdorf, Luis-Miguel Chevin, François Vasseur

**Affiliations:** 1 CEFE, Univ Montpellier, CNRS, EPHE, IRD, Montpellier, France; 2 LEPSE, Univ Montpellier, INRAE, Institut Agro Montpellier, Montpellier, France; 3 Plant Evolutionary Ecology, Institute of Ecology, Evolution and Diversity, Faculty of Biological Sciences, Goethe University Frankfurt, Frankfurt am Main, Germany; 4 CIRAD, UMR AGAP Institut, Montpellier, France; 5 UMR AGAP Institut, Univ Montpellier, CIRAD, INRAE, Institut Agro, Montpellier, France; 6 CEFE, Univ Montpellier, CNRS, EPHE, IRD, Univ Paul Valéry Montpellier 3, Institut Agro, Montpellier, France; 7 CEFE, Univ Montpellier, CNRS, EPHE, IRD, INRAE, Montpellier, France; 8 LBBE, Université Lyon 1, Lyon, France; 9 Department of Molecular Biology, Max Planck Institute for Biology Tübingen, Tübingen, Germany; 10 Institute for Bioinformatics and Medical Informatics, University of Tübingen, Tübingen, Germany; 11 Department of Integrative Biology, University of California Berkeley, Berkeley, California, United States of America; 12 Howard Hughes Medical Institute, University of California Berkeley, Berkeley, California, United States of America; 13 Plant Evolutionary Ecology, Institute of Evolution & Ecology, University of Tübingen, Tübingen, Germany; Indiana University, UNITED STATES OF AMERICA

## Abstract

Why do we observe some plant phenotypes but not others? The multivariate phenotypic space occupied by individuals or species often reveals both limits and phenotypes strikingly deviating from main syndromes. These observations are usually thought to indicate, respectively, inviable trait combinations and unique phenotypes adapted to specific environments. However, the evolutionary drivers underlying trait covariations often remain unclear. Here, we characterized the phenotypic space of *Arabidopsis thaliana* by comparing 713 wild accessions collected across the globe with 2,544 artificially-created recombinant individuals. This, combined with the detection of adaptive processes operating within species, allowed us to elucidate the roles of natural selection as a driver of phenotypic (co)variations within *A. thaliana*. We found that the phenotypic space of this species is constrained and driven by varying levels of divergent and stabilizing selection across different traits. Moreover, at the margins of the European geographic range, strong directional selection favored outlier phenotypes characterized by very late flowering and variation in a WRKY transcription factor gene. Genome analyses revealed that these extreme phenotypes may be explained by hybridization between ancestral and modern lineages of *A. thaliana*. Our findings demonstrate how interplays between population history and natural selection shape phenotypic diversity in a plant species.

## Abstract 2

Why do we observe some plant phenotypes but not others? This study uses investigation of the multivariate phenotypic space of a widely distributed plant species to reveal how interplays between population history and natural selection shape phenotypic diversity in Arabidopsis thaliana.

## Introduction

Explaining the exceptional diversity of organismal forms and functions is a fundamental aim of biology. This diversity, though large, is bounded in different ways across taxa, such that some combinations of trait values within and between species are rarely observed [1–5]. For instance, recent evidence suggests that the remarkable phenotypic diversity of vascular plant species covers a multivariate phenotypic space that is significantly smaller than expected from null distributions [5]. This result suggests that trait (co)variation in vascular plants is not completely random, but rather shaped by various types of constraints (*e.g.*, biophysical, genetic) and evolutionary processes. A key to understanding plant diversity is to investigate what shapes phenotypic (co)variation, which determines how phenotypes translate into functions and are targeted by natural selection [6].

Within species, directional selection drives phenotypic divergence between populations and can even promote the emergence of extreme phenotypes. In contrast, natural selection can also act by eliminating unfit genotypes and their associated trait combinations, thereby creating empty zones at the margins of the phenotypic space. Stabilizing selection is a pervasive force contributing to the absence of extreme phenotypes [7]. Most species are expected to undergo a combination of these two forms of selection: differences in phenotypic optima lead to divergent directional selection among populations, while stabilizing selection acts within populations once they reach their local optimum. The relevant question, therefore, concerns the relative magnitudes of these two components of selection in shaping phenotypic diversity [8]. By studying natural populations, we can only observe a limited fraction of a species’ potential diversity: the allelic combinations that have not been eliminated by chance or natural selection. A complementary approach is then to harness the standing genetic variation within a species, which can be substantial and harbor the potential to recreate phenotypes previously purged by selection. Producing recombinant individuals through controlled genetic crosses (F_2_s) offers a powerful approach to experimentally generate this potential diversity and compare it to the realized diversity observed in wild populations.

Meiotic recombination leads to the reshuffling of genetically based trait variation within species. Consequently, recombinant individuals have randomly segregating parental alleles that, in contrast to wild lines, do not result from past evolutionary processes [9–11]. They can then be used to estimate a null distribution of phenotypes that can be applied to test the role of natural selection as a driver of trait evolution and diversity [11]. One can notably assess how, and to what extent, the phenotypic space formed by distinct genotypes departs from that of the offspring generated from multiple crosses between them (through a multivariate approach, *e.g.*, [12]). If parental populations harbor a phenotype distribution that does not differ from the one expected by chance (*i.e.*, similar to the distribution observed in F_2_s), they have likely evolved without strong selective pressures [11]. If the opposite is true, the type(s) of selection that drove the phenotypes of the parental lines can be further investigated.

If F_2_ phenotypes fall outside the parental phenotypic range, a phenomenon called transgressive segregation, parental lines must have diverged from their common ancestor relying on distinct combinations of alleles across different loci (*i.e.*, not all “+” alleles in one parent and all “–” alleles in the other [13]). This condition, which is especially likely for polygenic traits [14,15], can be achieved either when parental phenotypes have evolved towards the same optimum or towards different optima [13]. However, transgressive segregation is thought to be more likely when the parents are not too phenotypically divergent [13,16], as it is otherwise less probable that the progeny will exceed the parental phenotypic range. Consequently, higher phenotypic variance in the progeny is thought to be more common under stabilizing selection combined with weak divergent selection. Conversely, strong directional selection that is divergent among populations should result in less transgressive segregation and a higher likelihood that the parental phenotypic range exceeds that of the F_2_s. However, these predictions remain poorly tested empirically [15] (but see [17,18] on the role of genetic distance and phenotypic differentiation).

When directional selection is strong enough, it can lead to phenotypes significantly deviating from mean trait combinations and thus at the margins of the phenotypic space (hereafter phenotypic outliers). However, phenotypic outliers can also emerge because of nonadaptive processes due to, *e.g.*, a lag in the response of certain populations to environmental changes [19]. For example, *Arabidopsis thaliana* has an evolutionary history marked by lineages that survived the Ice Age with minimal hybridization over time (relicts hereafter) and constitute apparent “outlier haplotypes” [20,21]. During the last glaciation, relict lineages were restricted to regions in southern Europe, from where the European continent was re-colonized after the Ice Age [21]. Most extant European populations originated from a genetic lineage that re-colonized all of Europe, likely from the Balkans, about 10,000 years ago [21]. Despite extensive hybridization that led to the loss of much of the relict genetic variation, a higher amount of relict introgression segments can still be found at both high and low latitudes in Europe (called refugia) [21]. One hypothesis for the maintenance of this genetic variation in extant populations is that it confers high adaptive value under specific environmental conditions [21–23]. However, it remains to be tested whether outlier haplotypes translate into outliers in the phenotypic space and whether natural selection can explain this pattern.

Here, we investigated to what extent selection has shaped vegetative and phenological trait (co)variations in 713 wild accessions of *A. thaliana* from a large geographic range. These accessions were grown in a common garden, together with 2,544 recombinant individuals produced by crossing 358 accessions that encompassed a broad range of genetic, geographic, and phenotypic variation [24]. We compared the realized phenotypic diversity within wild accessions to the potential phenotypic diversity arising from standing genetic variation through recombination. We specifically investigated (i) whether the phenotypic space of a widely distributed species is smaller than the overall distribution of recombinant individuals and whether this pattern varies across axes of phenotypic variation; (ii) the genetic bases of the phenotypic space and the relationship between phenotypic divergence in wild accessions and phenotypic segregation in the F_2_s; and (iii) the evolutionary drivers of phenotypic outliers among wild accessions. Our results demonstrate how population history and different types of selection can jointly shape the phenotypic diversity of a widely distributed plant species.

## Results

### Wild accessions occupy a smaller phenotypic space than recombinant F_2_s, but present phenotypic outliers

**Fig 1:**
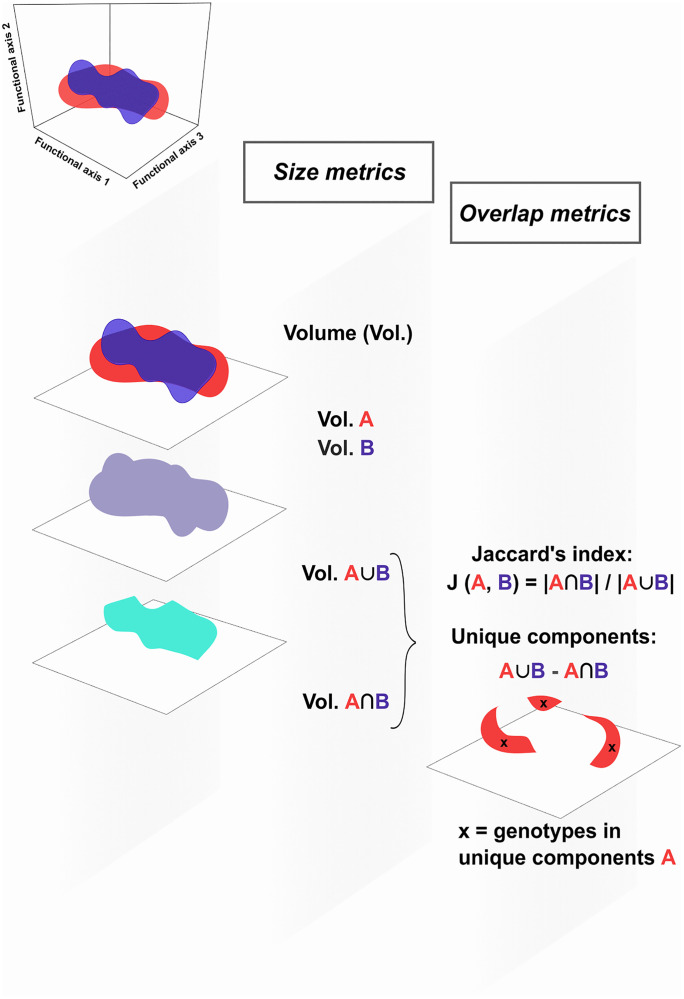
Analytical framework using the hypervolume approach. We quantified the realized and potential phenotypic spaces of **A*rabidopsis *thaliana** (here exemplified by groups A, in red, and B, in blue, respectively) using hypervolumes [12,25]. Hypervolumes are described through size (volume) and overlap metrics. Size informs about the extent of phenotypic variation, which we compared between groups using volume distributions obtained by resampling both groups to an equal number of observations. Overlap parameters are used to assess dissimilarity of phenotypic space position and unique components between groups. Dissimilarity was quantified through Jaccard’s similarity index, which is the proportion of overlap defined as the intersection of volumes A and B divided by their union. Unique components are quantified through the subtraction of the union of volumes A and B by their intersection. Within the unique components of one of the analyzed groups, here exemplified by A, we investigated the identity of the genotypes they were composed of (genotypes “x”).

**Fig 2:**
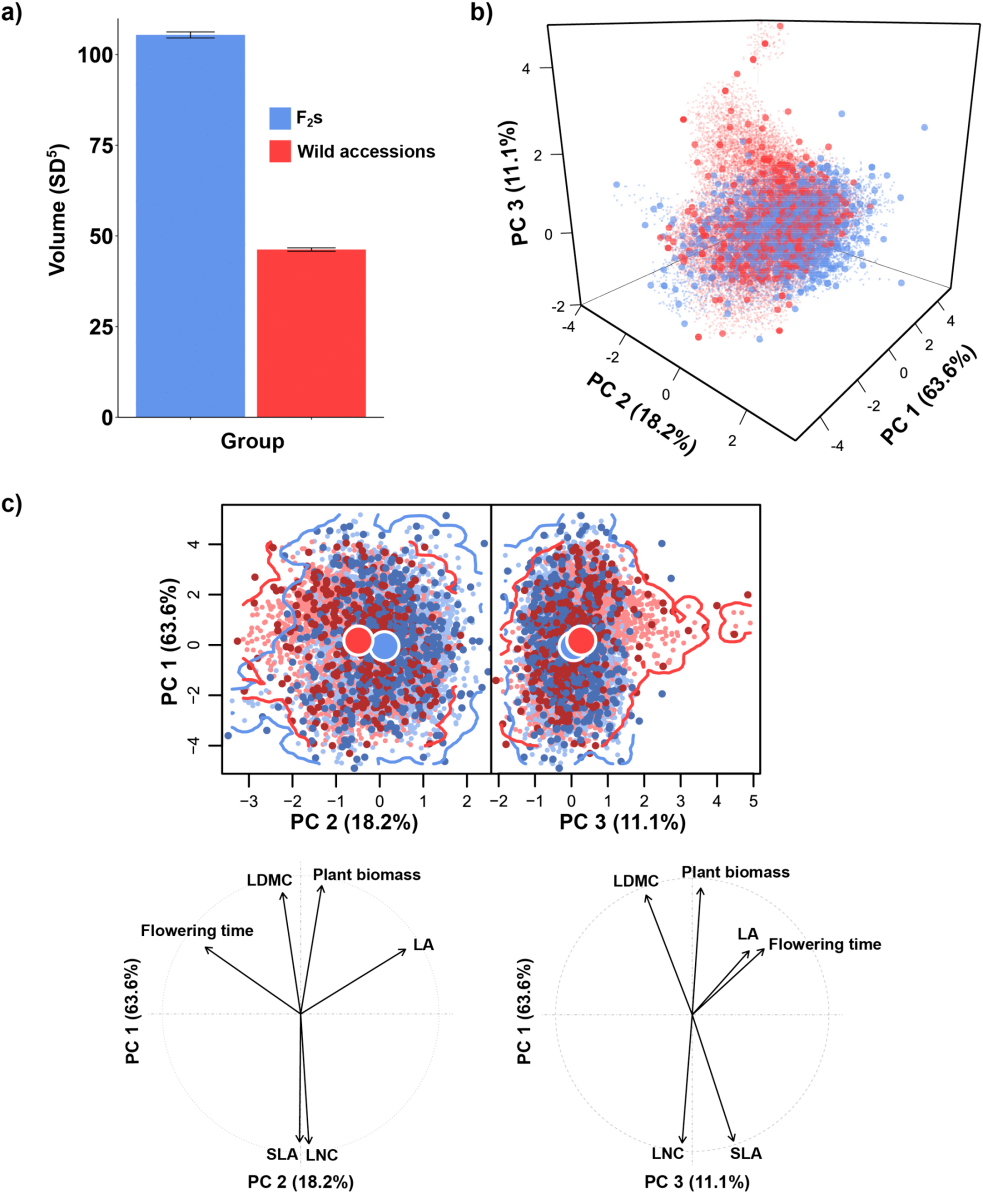
The phenotypic spaces of *Arabidopsis* *thaliana* F_2_s *vs.* wild accessions. The results shown correspond to one of the 10 hypervolume calculations. **a)** Hypervolume sizes (± standard errors), in units of standard deviation to power five (the number of trait dimensions used for hypervolume computation), for F_2_s (blue) *vs.* wild accessions (red). **b)** Trait hypervolumes based on the first three principal component analysis (PCA) dimensions (with opaque dots representing observed data points, and semitransparent small dots representing uniformly distributed random points generated by the algorithm used for hypervolume computation [27]). Hypervolumes with size equivalent to the mean of the 100 hypervolumes generated by resampling per group (F_2_s *vs.* accessions) are shown. **c)** Trait variation within the phenotypic spaces of *A. thaliana* F_2_s (blue) and wild accessions (red) considering the first three PCA dimensions of trait hypervolumes. Hypervolume centroids, observed data points, and uniformly distributed random points generated by the algorithm used for hypervolume computation [27] are depicted by large circles, opaque dots, and semitransparent small dots, respectively. Hypervolumes with size equivalent to the mean of the 100 hypervolumes generated by resampling per group (F_2_s *vs.* accessions) are shown. LA, leaf area; LDMC, leaf dry matter content; LNC, leaf nitrogen content; SLA, specific leaf area. The data underlying this figure can be found at: https://doi.org/10.48579/PRO/3LQH1M.

The phenotypic space of *A. thaliana* was assessed using the hypervolume approach [12,25] (Fig 1) on six traits measured on both F_2_ individuals (F_2_s) and wild accessions: flowering time; plant biomass; leaf area (LA), specific leaf area (SLA), leaf dry matter content (LDMC), and leaf nitrogen content (LNC). The selected traits encompass distinct plant strategies [5], including life-history traits (*i.e.*, flowering time and plant biomass), a size-related trait (*i.e.*, LA), and traits linked with the leaf economics spectrum (LES; *i.e.*, SLA, LDMC, and LNC), which depicts a trade-off between resource acquisition and conservation [26]. We used scores of five significant principal component analysis (PCA) dimensions to quantify the hypervolumes of F_2_s (*i.e.*, potential phenotypic space) and wild accessions (*i.e.*, realized phenotypic space). While controlling for differences in the sample sizes of F_2_s and wild accessions, we found that the hypervolume of F_2_s was over twice as large as that of wild accessions (Fig 2a), with about 42% overlap according to Jaccard’s index (S1 Fig). To describe trait variation within the phenotypic space of *A. thaliana*, we used the first three PCA dimensions, which together explained 92.9% of the variation (Fig 2b). PC1 explained 63.6% of the variation and largely reflected LES traits (*i.e.*, SLA, LDMC, and LNC) and plant biomass (Fig 2c), whereas PC2 and PC3 explained 18.2% and 11.1% of the variation and were mostly associated with LA and flowering time, respectively (Fig 2c). The variances of both PC1 and PC2 scores were significantly larger for the F_2_s compared with the wild accessions, but did not differ for PC3 (Breusch–Pagan test of homogeneity of variance: *P* < 0.05, *P* < 0.001, *P* = 0.24, respectively).

Not only did the phenotypic spaces of F_2_s and wild accessions differ in volume, but they also differed in their overlap metrics. Their degree of overlap depended on the PCA dimensions: overlap was high on PC1 but lower on PC2 and PC3 (Figs 2b, 2c, and S2). This pattern was mainly driven by the presence of 36 genotypes with combinations of trait values beyond those observed in F_2_s (Fig 2b and 2c, right and S1 Table). These were accessions forming the unique components of the accessions’ hypervolumes and hereafter referred to as “phenotypically unique accessions”. They were mainly characterized by very late flowering, the significance of which was confirmed by a sensitivity analysis. This analysis showed that removing flowering time led to a mean reduction of 66.6% of the volume of the phenotypic space of wild accessions compared to 57.3% of F_2_s (S3 and S4 Figs). Moreover, this procedure led to the detection of only four phenotypically unique accessions, confirming the importance of phenology in generating extreme variation.

Because the 713 accessions used to compute the phenotypic space of wild accessions included only 254 of the 358 parental (G_0_) accessions of the F_2_s, along with 459 additional accessions, we had to rule out any bias in the differences between the hypervolumes of F_2_s and accessions due to their genetic composition. We first found that the whole-genome genetic diversity (π) in the 254 G_0_ accessions and the other 459 analyzed accessions was highly correlated (S5 Fig). Next, we directly compared the hypervolume of the 254 G_0_ parental accessions with that of the F_2_s and still found that the latter was larger than the former (S6 and S7 Figs). Finally, we imputed the phenotypes of the 104 G_0_ accessions for which phenotypes had not been measured directly (358 minus 254 accessions) and found that F_2_s still presented a larger hypervolume when including these extra phenotypes (S8 and S9 Figs). Despite a large variation in the accuracy of imputation for some analyzed traits, flowering time was the trait that had the most accurate imputation (S2 Table). 12 and 35 phenotypically unique accessions out of the 36 initially recorded were still detected in the analysis with 254 accessions and in the analysis with imputed phenotypes, respectively (S7 and S9 Figs). In the former case, the reduction in the number of phenotypically unique accessions (66.7%, *i.e.*, 36–12) was comparable to the reduction in the number of analyzed accessions (64.4%, *i.e.*, 713–254).

### Divergent selection on traits influences the extent of the phenotypic space of wild accessions

**Fig 3:**
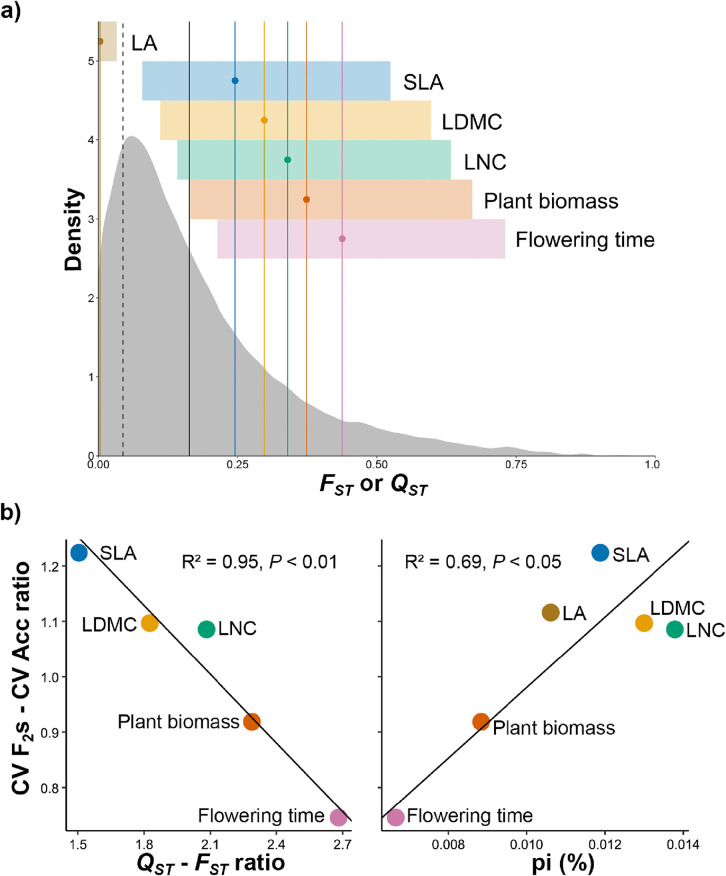
Phenotypic divergence in wild accessions of *Arabidopsis thaliana* and its relationship with the degree of transgressive segregation in F_2_s. **a)** Genetic variance of traits (*Q*_*ST*_ values, colored lines) and their credible interval (colored area) are compared with the neutral *F*_*ST*_ value (black solid line), which corresponds to the median of the distribution of significant *F*_*ST*_ values. The significance threshold was set at the 95th percentile of a null *F*_*ST*_ distribution (black dashed line), above which *F*_*ST*_ values were considered significant. **b)** Relationship between the genetic variance of traits (*Q*_*ST*_–*F*_*ST*_ ratio; all traits except LA, which did not show *Q*_*ST*_ within the range of significant *F*_*ST*_) and the degree of transgressive segregation (left), and between polygenicity (“pi” parameter) and the degree of transgressive segregation (right). Transgressiveness was measured as the ratio between the coefficient of variation (CV) of the traits in the F_2_s (CV F_2_s) and the CV of the traits in their parents (G_0_, CV Acc). Lines were fitted with Standardized Major Axis (SMA) regressions, and *R*^2^ denotes the coefficient of determination. LA, leaf area; LDMC, leaf dry matter content; LNC, leaf nitrogen content; SLA, specific leaf area. The data underlying this figure can be found at: https://doi.org/10.48579/PRO/3LQH1M and http://1001genomes.org/.

To understand the evolutionary mechanisms underlying lower variation in LES traits (*i.e.*, SLA, LDMC, and LNC) and higher variation in flowering time within wild accessions compared to F_2_s, we explored the genetic bases of the phenotypic space of the parental accessions of the F_2_s (G_0_). First, we performed Genome-Wide Association (GWA) for each trait and found no significant peak of SNP association (S10 Fig). For plant biomass, only one SNP in a noncoding genomic region was significantly associated (S10 Fig). We then performed polygenic GWA (Bayesian Sparse Linear Mixed Model [BSLMM, 28]) and found that, consistent with moderate to high heritability (“PVE” parameter, S3 Table; from 30.0% for LA to 84.4% and 91.1% for flowering time and LNC, respectively), all analyzed traits were highly polygenic, *i.e.*, explained by many SNPs of weak effect (“pi” parameter; S3 Table). We then tested the prediction that transgressive segregation for polygenic traits is more likely when the parents are not too phenotypically divergent (and have, thus, likely evolved under stabilizing selection combined with weak divergent selection). To this end, we first compared the degree of genetic variance of traits (*Q*_*ST*_) to neutral genetic variance (*F*_*ST*_) among G_0_ accessions to test for stabilizing *versus* divergent selection shaping each of the six analyzed traits. We found that LA had very low *Q*_*ST*_ (0.003), falling below the range of significant *F*_*ST*_ values (Fig 3a). In contrast, SLA, LDMC, LNC, plant biomass, and flowering time exhibited progressively higher *Q*_*ST*_ values relative to the median of the *F*_*ST*_ distribution (0.25, 0.30, 0.34, 0.37, and 0.44, respectively; Fig 3a). However, the difference was only significant (*Q*_*ST*_ > *F*_*ST*_) for life-history traits, *i.e.*, plant biomass and flowering time (Fig 3a). For the traits with *Q*_*ST*_ within the range of significant *F*_*ST*_ (all traits except LA), we examined the relationship between the ratio of *Q*_*ST*_ to *F*_*ST*_ and the degree of transgressive segregation (the ratio of the coefficient of variation (CV) of each trait in the F_2_s to the CV of the corresponding trait in their parents). We found higher transgressive segregation for traits with lower *Q*_*ST*_–*F*_*ST*_ ratio, *i.e.*, SLA, LDMC, and LNC (those associated with the LES; Fig 3b, left), and lower transgressive segregation for traits with higher *Q*_*ST*_–*F*_*ST*_ ratio, *i.e.*, flowering time and plant biomass (life-history traits; Fig 3b, left). This relationship was strong and significant (*R*^2^ = 0.95, *P* < 0.01; Fig 3b, left). Finally, we investigated the relationship between polygenicity (“pi” parameter) and the degree of transgressive segregation for all traits and found a moderate positive correlation between the variables (*R*^2^ = 0.69, *P* < 0.05; Fig 3b, right).

### Strong directional selection explains the presence of phenotypic outliers that are characterized by relict introgression segments

**Fig 4:**
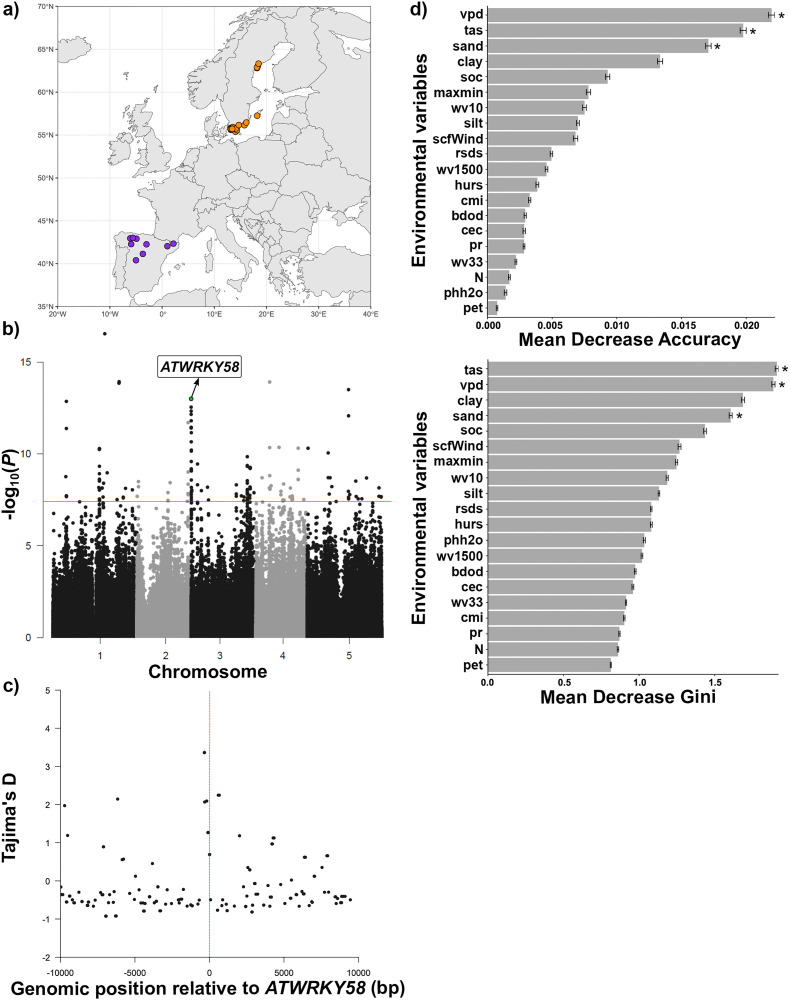
Characterization of phenotypically unique accessions. **a)** Map of the geographic origin of phenotypically unique accessions, showing those from Sweden (orange) and from Spain (purple). The map was created with data from Natural Earth via the R package *rnaturalearth* [30] (basemap shapefile: https://www.naturalearthdata.com/downloads/50m-cultural-vectors/). **b)** Manhattan plot showing SNPs that were significantly associated with the phenotypically unique *vs.* common accession classification. A SNP corresponding to the *ATWRKY58* gene, which was found to underlie the most prominent peak of diagnostic SNPs, is highlighted. The red line represents the significance threshold at *P* ≤ 0.05 with Bonferroni correction. **c)** Tajima’s *D* values were calculated every 100-bp window around the SNP that corresponded to the *ATWRKY58* gene (±10,000 bp). **d)** Relevance of different environmental variables in explaining the phenotypically unique *vs.* common accessions classification. Mean Decrease Accuracy (top) and Mean Decrease Gini (bottom) of the random forest models are presented. Asterisks represent selected variables through feature selection (Boruta) analysis. Bdod, bulk density; cec, cation exchange capacity; clay, proportion of clay particles; cmi, climate moisture indices; hurs, near-surface relative humidity; maxmin, the difference between mean daily maximum and minimum air temperature; N, total nitrogen content; pet, potential evapotranspiration; pHH_2_O, soil pH; pr, precipitation amount; rsds, surface downwelling shortwave flux in air; sand, proportion of sand particles; sfcWind, near-surface wind speed; silt, proportion of silt particles; soc, soil organic carbon content; tas, mean daily air temperature; vpd, vapor pressure deficit; wv10, volumetric water content at 10 kPa; wv33, volumetric water content at 33 kPa; wv1500, volumetric water content at 1,500 kPa. The data underlying this figure can be found at: https://doi.org/10.48579/PRO/3LQH1M, http://1001genomes.org/, https://www.chelsa-climate.org/datasets/chelsa_climatologies/, and https://files.isric.org/soilgrids/latest/data_aggregated/1000m/.

Since directional selection was strong for flowering time, we tested its role as a potential evolutionary driver of *A. thaliana* phenotypic outliers through further genomic and environmental analyses. We first found that phenotypically unique accessions were non-relicts from either Sweden (27 accessions mainly from South Sweden) or Spain (9 highland accessions mainly from North Spain, Fig 4a and S1 Table). Next, we identified SNPs that significantly differentiated these unique accessions from accessions with phenotypic syndromes that were common in the studied groups (Fig 4b). The most prominent peak of diagnostic SNPs for the phenotypically unique accessions was in chromosome 3 and included variants in a WRKY transcription factor gene that has been linked to senescence and stress response (*ATWRKY58* [29], Fig 4b). We also found that *ATWRKY58* coincided with an increase in Tajima’s D values compared to the surrounding genomic sequences (Fig 4c). Finally, based on the environmental analyses, the variables vapor pressure deficit (VPD), mean daily temperature, and sand content were shown to play major roles in explaining the differences between phenotypically unique and common accessions (Fig 4d). They were the variables that mostly decreased accuracy and Gini index in the random forest models (mean OOB = 0.32) and that were selected in the feature selection (Boruta) analysis (Fig 4d). VPD and mean daily temperature were significantly lower for phenotypically unique relative to common accessions (**P* *< 0.001 and **P* *< 0.01, respectively; S11 Fig). Soil sand content, in turn, was significantly higher for phenotypically unique relative to common accessions (**P* *< 0.01, S11 Fig).

Using published data [21], we found relict introgression segments in phenotypically unique accessions. Spanish phenotypically unique accessions, from locations close to the oldest relict refugia [20], had more introgressions than Swedish phenotypically unique accessions (mean ± SE: 163.00 ± 17.93 and 74.52 ± 3.97, respectively) and presented an intermediate number of relict haplotypes between Spanish relicts (geographically closer to Spanish phenotypically unique accessions and possessing high frequency of relict haplotypes, mean ± SE: 737.23 ± 34.43) and French accessions (phenotypically common accessions used before as a non-relict reference due to their very low level of relict haplotypes [21], 52.84 ± 2.37, S12 Fig). We confirmed the importance of relict introgressions in phenotypically unique accessions by comparing vegetative, phenological, and reproductive traits between phenotypically unique, Spanish relict, and French accessions. We found that phenotypically unique accessions were more similar to Spanish relicts than to French accessions, mostly regarding plant biomass, but also LA and LNC (S13a Fig). For flowering time, although phenotypically unique accessions had longer flowering time than both Spanish relicts and French accessions, this difference was smaller relative to the former than to the latter (S13b Fig). Phenotypically unique accessions also generally had lower reproductive performance, either with smaller fruit length or reduced fertility compared to French accessions, but not to relicts (S13c Fig).

## Discussion

Here, we combined phenotypic, genomic, environmental, and biogeographic information about *A. thaliana* to cast light on the evolutionary determinants that shaped its phenotypic diversity. Our results revealed that wild accessions of *A. thaliana* generally occupied a reduced phenotypic space compared to F_2_ individuals (F_2_s), suggesting that the phenotypic space of *A. thaliana* is constrained. However, at the margins of the European geographic range, strong directional selection acted by favoring phenotypic outliers with relict genetic variation.

The genetic mechanisms that may explain F_2_ phenotypes falling outside the phenotypic range of parental populations rely on the phenomenon of transgressive segregation [13]. In accordance with previous predictions [13,16], we found that, for highly polygenic traits such as the six traits analyzed here, the degree of transgressive segregation decreases with the strength of divergent selection (estimated through *Q*_*ST*_–*F*_*ST*_ ratio). Accordingly, lower differentiation in parental accessions was shown to be associated with higher transgressive segregation. Furthermore, polygenicity was positively associated with the degree of transgressiveness, corroborating this type of genetic architecture as key for the emergence of transgressive segregation [15]. Taken together, these observations explain why relatively more polygenic traits for which accessions had genetic variance not significantly higher than the neutral expectation (such as traits that underlay the main axis of variation of the phenotypic space of *A. thaliana*: LES, *i.e.*, SLA, LDMC, and LNC) would more likely fall within the phenotypic range of the F_2_s. Conversely, relatively less polygenic and highly differentiated life-history traits (among accessions), such as flowering time, would more likely fall outside the phenotypic range of the F_2_s, as observed here.

In line with our findings, flowering time has been shown to be under pervasive directional selection in *A. thaliana* [31–34], while LES traits have been found to be differentiated among wild accessions but without a significant signal of directional selection [35]. Beyond these contrasting patterns, plant biomass showed a more nuanced trend: whereas it was associated with the LES dimension (PC1) of the phenotypic space of *A. thaliana*, the *Q*_*ST*_–*F*_*ST*_ analysis suggested that it is under directional selection and intermediary in the gradient of phenotypic differentiation of parental lines *versus* transgressive segregation of the progeny. This is consistent with previous findings showing that plant biomass in *A. thaliana* can be correlated with both flowering time and LES traits [35,36] and suggests that variation in this trait is shaped by a combination of different forces: diverging and stabilizing because of its link with phenology and LES traits, respectively.

Stabilizing selection acting on phenotypic variation is inherently difficult to detect [8]. This is likely due to the fact that, in nature, fluctuating environments keep populations away from the fitness optimum [37]. To circumvent this difficulty, comparing the phenotypic distribution of F_2_s *versus* their parents is a powerful approach, as higher phenotypic variance in the F_2_s indicates forces limiting the phenotypic range of the parental lines [11]. However, among the traits explaining the major axes of variation in the phenotypic space of *A. thaliana*, we could confirm through *Q*_*ST*_–*F*_*ST*_ analyses the potential action of stabilizing selection on only one trait: LA (though the *F*_*ST*_ values for this trait fell below the chosen significance threshold). Another mechanism that can also explain a part of the phenotypic variation between wild accessions and F_2_s is overdominance (wherein recessive deleterious mutations are masked at loci that are divergent between parental populations due to heterozygosity [38]), as approximately half of the genome of F_2_s is heterozygous. However, its effect is primarily on mean values (overlap metrics), as overdominance is not expected to contribute to variance [38] (volume and PC variance). Moreover, it is unlikely that there is much selection on heterozygous allelic combinations in *A. thaliana* due to its selfing nature [39]. Finally, it is important to highlight that genetic constraints (*i.e.*, linkage disequilibrium and pleiotropy) may interact with stabilizing selection and divergent selection to explain the observed limits to phenotypic variation, since trait correlations were generally the same for parental accessions and F_2_s (S14 Fig). Pleiotropic alleles have been previously found to underlie leaf economics traits in wild accessions of *A. thaliana* [23]. However, further research is required to clarify the interplay between natural selection and genetic constraints acting on multiple traits within this plant species.

Flowering time was found to be under strong directional selection, promoting phenotypic outliers in the phenotypic space of *A. thaliana* characterized by very late flowering. Most studies have revealed pervasive selection for earlier, rather than delayed flowering—likely as a response to climate change [31–34]. Studies demonstrating selection for late flowering have revealed that it may be driven by cold conditions [31,40–43] and late frost [44]. In accordance with these results, we identified some *A. thaliana* genotypes (phenotypically unique accessions) that possibly resulted from directional selection for very late flowering in cold and humid regions with high latitude (Sweden) and high altitude (Spain). We also found that balancing selection (positive Tajima’s D) seems to underlie the gene that was mostly linked with the extreme phenotype of phenotypically unique accessions, *i.e.*, a WRKY transcription factor gene (*ATWRKY58*). This may indicate that variation in this gene is key for the adjustment of flowering time to changing conditions in *A. thaliana*. WRKY transcription factor genes have been previously associated with response to stress in *A. thaliana* [45], and their role in regulating senescence and the response to abiotic and biotic factors has been described across various plant species [46]. However, only more recently have the effects of the WRKY family on flowering time been highlighted [47–49]. For instance, distinct genes in this family influence flowering differently by either advancing or delaying it through hormone-induced pathways [50].

Beyond pinpointing specific genetic variation that underpins phenotypic outliers, we found that phenotypically unique accessions were not relicts *stricto* *sensu*, but that their genomes had introgressed chunks of relict genomes. This is in accordance with their very different geographic origins (Sweden or Spain), which are linked with the evolutionary history of *A. thaliana* and the presence of relict variation in refugia across both high and low latitudes in Europe [21,51]. Moreover, phenotypically unique accessions presented life-history (*i.e.*, plant biomass and flowering time) and reproductive traits (*i.e.*, fruit length and fertility) that were more similar to relicts than to French accessions. Previous studies have already shown that relictual genetic variation was associated with life-history transitions, such as flowering and senescence [23,52]. However, little is known about how these transitions relate to functional traits and fitness [43,53].

Also analyzing a Swedish genotype of *A. thaliana*, Postma and Ågren [43] showed that larger rosette size led to later flowering and was generally positively correlated with survival and the number of fruits produced per plant. Although the phenotypically unique accessions found here had high plant biomass, they generally presented low fertility relative to phenotypically common accessions. This is in accordance with previous studies that found late-flowering genotypes to have lower seed production than early-flowering genotypes [54,55]. However, it is possible that phenotypically unique accessions would exhibit higher fertility under colder growth conditions (such as those in Postma and Ågren’s [43] experimental setup), a hypothesis that can be tested in future studies. All these contrasting results underscore the fact that genotype-phenotype relationships and their fitness consequences are context-dependent [31,56]. We therefore hypothesize that humid and cold conditions, along with a sandy soil texture, could modulate the expression of regulatory genes that indirectly impact flowering transition to allow the maintenance of phenotypic outlier accessions.

## Conclusions

We tested whether the phenotypic space of a widely distributed plant species is constrained by natural selection, and whether and how phenotypic outliers have emerged within this space. We revealed that the phenotypic space of thousands of genetic crosses (F_2_s) was significantly larger than that of wild accessions of *A. thaliana*. This analysis, combined with the investigation of differentiation patterns within accessions, suggested that the phenotypic space of this species is constrained and that both divergent and stabilizing selection influence its extent. Despite these limits, phenotypic outliers emerged at the margins of the species’ distribution in Europe, in admixed populations with introgressed relict haplotypes and genetic variation that probably evolved by strong directional selection in response to particular environments. As the evolutionary history of *A. thaliana* has demonstrated, relict genomic variation may be crucial for its adaptation to climate change, and we advocate for further investigation of populations with relict introgressions. Understanding the drivers of trait (co)variation and the conditions for local adaptation is imperative if we want to predict the impacts of climate change on plant phenotypes and ecology.

## Materials and methods

### Plant material

We used two sets of *A. thaliana* genotypes, based on an initial selection of accessions covering a wide range of genetic, geographic, and phenotypic variation, thereby preventing ascertainment bias (a general caveat of trait-based tests of selection [11]). The final genotype sets included:

(i) 3,150 recombinant individuals (F_2_s) from 350 crosses among 358 wild accessions (selected as explained above [24]), which we used to experimentally generate a null distribution of phenotypes (potential phenotypic space);
(ii) 713 wild accessions, including 254 parental (G_0_) accessions of the F_2_s and 459 additional accessions—selected from Exposito-Alonso and colleagues’ [51] list of accessions (filtered from the 1,001 Genomes Project [20] for maximizing geographic coverage and genetic diversity of *A. thaliana*)—, which we used to experimentally generate an observed distribution of phenotypes (realized phenotypic space).

### Experimental setup

We conducted a completely randomized outdoor common garden experiment between February and July 2021 at the Centre d’Ecologie Fonctionnelle et Evolutive (CEFE), Montpellier, France. We sowed plants in pots of 0.08 L filled with a sterilized soil mixture of 50% river sand, 37.5% calcareous clay soil from the experimental field at CEFE, and 12.5% blond peat moss. A total of 3,150 genetically different F_2_ individuals (F_2_s) were sown at the beginning of the experiment by randomly selecting seeds from a pool of around 2,570 seeds per F_2_ cross. These plants were randomly distributed across three spatial blocks, with no replicates per genotype. For wild accessions, we initially sowed one individual per accession per block (three replicates per genotype), for a total of 2,139 wild-accession individuals at the beginning of the experiment. Each block had an independent subirrigation system, and plants were irrigated three times per week until the beginning of flowering. Details of the setup have been described in Przybylska and colleagues [57].

### Trait measurements

To assess both the potential (F_2_s) and realized (wild accessions) phenotypic spaces of *A. thaliana*, we recorded six traits for each individual that reached the flowering stage (first flower at anthesis): one trait related to phenology (plant age at flowering, hereafter flowering time), four traits related to plant resource acquisition and conservation (LA, LDMC, LNC, and SLA), and one trait related to plant growth (rosette biomass, hereafter plant biomass). From the 2,139 individuals of wild accessions and the 3,150 F_2_ individuals initially sown, 1,730 and 2,544, respectively, reached the flowering stage and could be measured.

Our trait measurements followed established protocols [57]. Briefly, after plants had been irrigated for at least 2 h to ensure leaf rehydration [58,59], we selected a fully developed leaf exposed to sunlight for leaf trait measurement [59]. We determined LA through image analysis, using ImageJ 1.53k software [60], and estimated LNC through near infrared spectroscopy, according to methods described before [57,61]. Leaves that were smaller than the area of the spectrometer probe could not be analyzed. As such leaves represented less than 10% of the data, we conducted “NA” imputation using the R package *missMDA* [62]. Leaf and rosette dry weights were recorded after subjecting the samples to 60 °C for 3 days. Flowering time was converted to growing-degree days as the daily cumulation of Celsius degrees (°C) from sowing until the flowering date (for details, see [57]).

### Multivariate analyses

Before computing the phenotypic space of *A. thaliana*, we had to account for the significant block effects on trait variation. To do this similarly for both groups (F_2_s, without genotype replication, and wild accessions, with genotype replication), we first calculated least-square estimates of the block effect on each trait of the accession group (the group for which genotype and block effects could be disentangled), and we took the estimate for the first block as a reference. Next, we calculated the estimate deviation of the two other blocks relative to the reference [63]. Finally, we corrected each trait value (for both F_2_s and wild accessions) in the second and third blocks by their respective deviation to the first block. After this procedure, we calculated the average values of each trait for each wild accession. We log_10_-transformed flowering time and SLA, and square root-transformed LNC and plant biomass to meet the assumptions of parametric analyses.

To compute the potential and realized phenotypic spaces of *A. thaliana*, we performed PCA on centered and standardized trait values of F_2_s and wild accessions, respectively. Using scores of five significant PCA dimensions (according to a sequential Bonferroni procedure; R package *ade4*, *testdim* function [64]), we calculated separate hypervolumes (*i.e.*, the parts of *n*-dimensional Euclidean spaces, composed of independent axes of variation and occupied by the observed phenotypic values) for the two types of plant material. Such hypervolumes can be characterized by geometrical metrics, such as size (volume) and overlap [12,25,27] (Fig 1), and have so far mainly been used in ecology to compare the phenotypic spaces of different species or groups of species [5,12,25,27]. Hypervolume sizes informed us about the extent of the potential and realized phenotypic spaces in *A. thaliana*. Overlap parameters, in turn, allowed us to assess dissimilarities (in terms of position) between the potential and realized phenotypic spaces, as well as to identify wild accessions with trait values not observed in F_2_s (unique phenotypes at the margins of the realized phenotypic space, *i.e.*, phenotypic outliers).

We calculated hypervolumes using the Support Vector Machine algorithm of the R package *hypervolume*, which is suitable for considering outliers during hypervolume estimation [27]. As differences in sample size can impact hypervolume comparisons [25], we resampled both F_2_s and wild accessions to an equal number of 500 observations. This resampling was conducted 100 times using the *hypervolume_resample* function of the R package *hypervolume* [12,25,27]. This allowed us to obtain standard errors for the size (*i.e.*, volume) of hypervolumes (based on 100 hypervolumes estimated for F_2_s and another 100 estimated for wild accessions) and a distribution of overlap parameters (based on the 10,000 possible combinations of 100 hypervolumes of F_2_s and wild accessions). The first overlap parameter obtained was Jaccard’s similarity index, which is defined as J (*A*, *B*) = |*A*∩*B*| / |**A∪B**|, with *A* and *B* equaling the hypervolumes in groups A and B, respectively, and || denoting a cardinal (size of a group, Fig 1). To put it simply, J (*A, B*) in our study was the proportion of the total phenotypic hypervolume that was shared between F_2_s and wild accessions. The second overlap parameter was unique components, that is, the size of the nonoverlapping parts between hypervolumes (|*A*∪*B*|–|*A*∩*B*|, Fig 1). We used this parameter to detect unique phenotypes at the margins of the realized phenotypic space (*i.e.,* phenotypic outliers). We used the function *hypervolume_inclusion_test* of the R package *hypervolume* [12,25,27] to test which accessions were present in each one of the 10,000 accessions’ unique components produced (Fig 1). We then selected the accessions that contributed to 95% of the entire set of accessions in unique components, here called phenotypically unique accessions. Because hypervolume delineation involves the generation of uniformly distributed random points in the surroundings of sets of data points [27], the results of the hypervolume calculations explained here may vary slightly between runs. Therefore, we repeated 10 times the calculation of the hypervolumes and overlap parameters. The final list of phenotypically unique accessions was defined based on the accessions that were present in at least six of the 10 generated lists of phenotypically unique accessions. Using this final set of accessions, we conducted further analyses to understand the molecular (genomic) and evolutionary mechanisms that could explain their unique functional traits (see the following three sections).

As mentioned in the “Plant material” section, 254 parental (G_0_) accessions of the F_2_s, along with 459 non-parental accessions, were phenotyped to generate the hypervolume of wild accessions, whereas 104 G_0_ accessions were not phenotyped and were therefore missing in this analysis. This could generate systematic differences between the hypervolumes of accessions and F_2_s. To ensure that it was not the case, we performed three additional analyses. First, we tested if the 254 analyzed G_0_ accessions were systematically different from the other 459 accessions. For that, we compared the genetic diversity (*π*) between these two groups using VCFtools (0.1.16) [65]. For each group of genotypes, we assembled SNP data from the 1,001 Genomes Project [20] (http://1001genomes.org/), removing SNPs with lower than 5% minor allele frequency (MAF) and missing data over 5%. Then, we calculated *π* for every 10,000 bp window of the genome and verified the correlation of these values between groups of genotypes using the function *sma* of the R package *smatr* [66]. Second, we reran the hypervolume analyses described in the previous paragraph, this time including only the 254 G_0_ accessions in the computation of the hypervolume of wild accessions. Third, using the genetic and phenotypic information of the 713 analyzed wild accessions, we imputed the phenotype of the 104 G_0_ accessions for which phenotypes had not been measured directly. To this end, we first removed, using VCFtools (0.1.16) [65], SNPs with lower than 1% MAF and missing data over 5%, restricted to a list of the 817 (713 + 104) analyzed accessions. Then, using the output of a BSLMM performed with GEMMA 0.98.3 [28,67] and setting the default number of 1,000,000 iterations and 100,000 burn-in steps, we imputed the six missing traits of the 104 G_0_ accessions. BSLMM computation and phenotypic imputation were conducted per trait and repeated 100 times. Imputed trait values were averaged per accession and incorporated into the phenotypic dataset of wild accessions. With this dataset now composed of six traits of 817 accessions *versus* 2,544 F_2_s, we reran the hypervolume analyses described in the previous paragraph. To verify the accuracy of the performed phenotypic imputation, we randomly selected, 100 times, 91 accessions from the 713 for which we had trait measures (a proportion of imputed phenotypes that was equivalent to the 104 imputed phenotypes out of 817), and we imputed their values for the six analyzed traits using the same procedure described before, but restricted to the original list of 713 accessions. We averaged the trait imputations per accession and verified their correlation with the observed values using the function *sma* of the R package *smatr* [66].

Finally, because the PCA analysis indicated flowering time as the main trait underpinning phenotypically unique accessions, we performed a sensitivity analysis to test for the influence of this trait. We removed flowering time from the trait dataset and reran the PCA comparison and the hypervolume analyses of 713 accessions *versus* 2,544 F_2_s, according to the same procedures described before.

### Genomic analyses

To shed light on the possible types of selection acting on different traits in *A. thaliana*’s wild accessions, we explored the genetic bases of the phenotypic space of the 254 G_0_ accessions. We first performed GWA for each of the six analyzed traits. To this end, we obtained *A. thaliana*’s SNPs from the 1,001 Genomes Project dataset [20] (http://1001genomes.org/) and used VCFtools (0.1.16) [65] to filter them according to a MAF of 5% and maximum missing data of 5%, restricted to a list of the 254 accessions here analyzed. This procedure returned a set of 546,179 SNPs, which was then used in PLINK 2.0 [68] and GEMMA 0.98.3 [67] to perform the GWA, while controlling for population structure. Next, we performed polygenic GWA. Applying the same parameters described above to filter SNPs, we used PLINK 2.0 [68] and GEMMA 0.98.3 [67] to associate them with each analyzed trait through a BSLMM [28]. We adopted the default number of iterations and burn-in steps (*i.e.*, 1,000,000 and 100,000, respectively) and ran the BSLMM model 100 times for each trait separately, averaging the results across runs. While controlling for population structure [28], the BSLMM model estimates “chip heritability” (*i.e.*, the proportion of the phenotypic variance explained by the analyzed set of SNPs, “PVE” hyperparameter) and the proportion of SNPs presenting a larger effect (which we used to infer polygenicity, “pi” hyperparameter).

Since all analyzed traits were polygenic, we tested if transgressive segregation is more likely when the parents are not too phenotypically divergent. For that, we used the classification of *A. thaliana* accessions in genetic groups from the 1,001 genomes dataset, excluding the “admixed” group [20] (http://1001genomes.org/), and calculated *Q*_*ST*_ for each analyzed trait as the among-group phenotypic variance divided by the total phenotypic variance. This analysis was performed for 210 G_0_ accessions (as 44 belonged to the “admixed” group) by fitting multi-response generalized linear mixed models, which also generate 95% credible intervals around the *Q*_*ST*_ values (R package *MCMCglmm* [69]). We then performed *Q*_*ST*_ *versus F*_*ST*_ comparisons. To this end, we created a distribution of significant Weir and Cockerham’s *F*_*ST*_ calculated per intergenic SNP (125,413 significant SNPs out of 150,479 SNPs), using PLINK 1.9 [70], and defined its median as the neutral *F*_*ST*_ value against which *Q*_*ST*_ values were compared. We calculated *F*_*ST*_ values based on the group classification described above and assessed their significance by randomly permuting group labels 1,000 times to generate a null distribution. The 95th percentile of this distribution was defined as the threshold above which *F*_*ST*_ values were considered significant [35]. Finally, for each trait, we calculated the degree of transgressive segregation in the F_2_ lines by dividing the CV of each trait in these lines by the CV of the corresponding trait in the G_0_ accessions. We then regressed this ratio against the ratio of *Q*_*ST*_ to the neutral *F*_*ST*_ value and the polygenicity metric (“pi” hyperparameter). We performed regression analysis using the *sma* function from the *smart* R package [66].

To assess the genomic mechanisms that could explain the differences between phenotypically unique and common accessions, we used the whole pool of wild accessions to perform GWA for the phenotypically unique *versus* common accessions classification. For that, using VCFtools (0.1.16) [65], we first filtered SNPs according to a MAF of 1% and maximum missing data of 5%, restricted to a list of the 713 accessions here analyzed. This procedure returned a set of 1,330,670 SNPs, which was then used in GEMMA 0.98.3 [67] to perform the GWA, while controlling for population structure. To assess the underlying genes and their functions, we used The *Arabidopsis* Information Resource (TAIR, https://www.arabidopsis.org/). Finally, still applying the same parameters in VCFtools (0.1.16) [65], we calculated Tajima’s D metric every 100-bp window to test for signatures of selection.

### Environmental analyses

To test if environmental variation explained the functional trait differences between phenotypically unique and common accessions, potentially leading support to natural selection as a driver of phenotypic outliers, we also analyzed the environments of origin of the wild accessions. For that, we extracted environmental variables from global climatic and pedologic layers using GPS coordinates, obtained from the 1,001 Genomes Project dataset [20] (http://1001genomes.org/), and the R package *terra* [71]. Climatic layers were obtained from CHELSA 2.1 climatologies across 1981–2010 [72,73], in 30 arc-second resolution. We extracted the following climatic variables, monthly predicted between October and June and averaged across these months (the period of the year in which *A. thaliana* generally grows and sets seeds): climate moisture indices, mean daily air temperature, near-surface relative humidity, near-surface wind speed, potential evapotranspiration, precipitation amount, surface downwelling shortwave flux in air, the difference between mean daily maximum and minimum air temperature, and VPD. Pedologic layers were obtained from ISRIC—World Soil Information [74], in 250 m aggregated to 1 km resolution, for 5 cm soil depth. We extracted the following soil variables: bulk density, cation exchange capacity, proportion of clay particles, proportion of sand particles, proportion of silt particles, total nitrogen content, soil organic carbon content, pH, volumetric water content at 10 kPa, volumetric water content at 33 kPa, and volumetric water content at 1,500 kPa. To evaluate the association of different environmental variables with phenotypically unique *versus* common accessions, we first centered and scaled all environmental variables. Since the classification “phenotypically unique *vs.* common” was highly unbalanced, we first resampled phenotypically common accessions 10,000 times to the sample size of phenotypically unique accessions and then conducted a random forest analysis on each set of data (using the R package *randomForest* [75], with ntree = 2,000). We computed mean variable importance and mean error rate of classification (out-of-bag estimate of error, OOB) across runs. To confirm the most relevant environmental variables for the phenotypically unique *versus* common classification, we used feature selection with each set of data (R package *Boruta* [76]), calculating the frequency of selection or rejection for each feature. We considered features as relevant when they were selected with a minimal frequency of 60%.

### Extended comparisons for phenotypically unique *versus* common accessions

Phenotypically unique accessions did not belong to the relict genetic group (S1 Table), but this did not eliminate the possibility that their genomes have introgression segments from relicts. To test this possibility, we first examined published data of relict haplotypes among accessions of the 1,001 Genomes Project [20]. Then, we characterized trait similarity of phenotypically unique relative to Spanish relict (geographically closer to Spanish phenotypically unique accessions and possessing a high level of relict haplotypes [21]) and French common accessions (used before as a non-relict reference due to their very low level of relict haplotypes [21]). The country of origin and the genetic group (which defined the classification as relict or non-relict) of the accessions were obtained from the 1,001 Genomes Project dataset [20] (http://1001genomes.org/). To characterize trait similarity, we used not only the phenological and vegetative traits explained above, but also reproductive traits measured during the experiment: fruit (*i.e.*, silique) length and number, inflorescence length, and seed mass. These traits were not measured in the same individuals analyzed for phenological and vegetative traits, because they needed to be assessed in a different phenological stage: fruit maturation (instead of flowering). Moreover, due to experimental constraints, we could not assess reproductive traits in every 713 accessions selected for the phenotypic space analyses. Because of that, we randomly selected 529 wild accessions from the initial pool, and we sowed them in three additional replicates (529 accessions × 3 blocks [57]). Because of the random selection of accessions and the fact that 495 out of the 529 initial accessions reached fruit maturation and could be measured, only 17 phenotypically unique accessions (14 from Sweden and 3 from Spain) were included in this sampling. Traits were measured as described in Przybylska and colleagues [57]. Inflorescences were cut at their rosette base and then photographed in their entirety. Except for seed mass, we estimated all reproductive traits through image analysis using ImageJ 1.53k [60]. Fruit length was measured as the mean length of three randomly selected mature fruits per plant. Fertility was estimated through the product of mean fruit length and fruit number [77,78]. To measure seed dry mass, we dried inflorescence stems at ambient temperature and weighed around 30 seeds per plant for an estimation of individual seed mass.

Using all the traits available for the 495 accessions mentioned above (*i.e.*, phenological, vegetative, and reproductive traits), we calculated least-square means with block as a random factor. We then compared trait values of phenotypically unique accessions with those of Spanish relicts and French common accessions, using nonparametric tests (Kruskal–Wallis and Dunn test with Holm’s correction for *post hoc* comparisons).

All statistical analyses were carried out in R 4.4.0 [79].

## Supporting information

S1 Table List of the phenotypically unique accessions. Information about their genetic group and country of origin, extracted from the 1,001 Genomes Project dataset (http://1001genomes.org/), is provided.
(DOCX)

S2 Table Accuracy of trait imputation for the 104 G_0_ accessions for which phenotypes had not been measured directly. *R*^2^ denotes the coefficient of determination. LA, leaf area; LDMC, leaf dry matter content; LNC, leaf nitrogen content; SLA, specific leaf area.
(DOCX)

S3 Table “Chip heritability” (“PVE”) and the proportion of SNPs presenting a larger effect (“pi”) on traits of wild accessions. LA, leaf area; LDMC, leaf dry matter content; LNC, leaf nitrogen content; SLA, specific leaf area.
(DOCX)

S1 Fig Jaccard’s index distribution for the comparison between hypervolumes obtained for F_2_s and wild accessions. The distribution is based on 10,000 comparisons between resampled hypervolumes for each group. The result shown corresponds to one of the 10 hypervolume calculations. The data underlying this figure can be found at: https://doi.org/10.48579/PRO/3LQH1M.
(TIFF)

S2 Fig Trait variation within the phenotypic spaces of F_2_s (blue) and wild accessions (red) of *Arabidopsis* *thaliana*, based on nine hypervolume recalculations. Variation for the first three PCA dimensions of trait hypervolumes is presented. Hypervolume centroids, observed data points, and uniformly distributed random points generated by the algorithm used for hypervolume computation [27] are depicted by large circles, opaque dots, and semitransparent small dots, respectively. Hypervolumes with size equivalent to the mean of the 100 hypervolumes generated by resampling per group (F_2_s *versus* accessions) are shown. The data underlying this figure can be found at: https://doi.org/10.48579/PRO/3LQH1M.
(ZIP)

S3 Fig Hypervolume sizes for F_2_s (blue) *versus* wild accessions (red) when excluding flowering time from the analyses. Size is represented in units of standard deviation to power five (the number of trait dimensions used for hypervolume computation). The result shown corresponds to one of the 10 hypervolume calculations. Bars denote SE. The data underlying this figure can be found at: https://doi.org/10.48579/PRO/3LQH1M.
(TIFF)

S4 Fig Trait variation within the phenotypic spaces of F_2_s (blue) and wild accessions (red) of *Arabidopsis thaliana*, based on 10 hypervolume calculations without flowering time. Variation for the first three PCA dimensions of trait hypervolumes is presented. Hypervolume centroids, observed data points, and uniformly distributed random points generated by the algorithm used for hypervolume computation [27] are depicted by large circles, opaque dots, and semitransparent small dots, respectively. Hypervolumes with size equivalent to the mean of the 100 hypervolumes generated by resampling per group (F_2_s *versus* accessions) are shown. LA, leaf area; LDMC, leaf dry matter content; LNC, leaf nitrogen content; SLA, specific leaf area. The data underlying this figure can be found at: https://doi.org/10.48579/PRO/3LQH1M.
(ZIP)

S5 Fig Correlation between the whole-genome genetic diversity (π) of the 254 parental (G_0_) accessions of the F_2_s and the other 459 accessions analyzed in this study. Each point represents one genomic region. The line was fitted with Standardized Major Axis (SMA) regressions, and *R*^2^ denotes the coefficient of determination. The data underlying this figure can be found at: https://doi.org/10.48579/PRO/3LQH1M and http://1001genomes.org/.
(TIFF)

S6 Fig Hypervolume sizes for F_2_s (blue) *versus* wild accessions (red) when analyzing only the 254 G_0_ accessions that were directly phenotyped in this study. Size is represented in units of standard deviation to power five (the number of trait dimensions used for hypervolume computation). The result shown corresponds to one of the 10 hypervolume calculations. Bars denote SE. The data underlying this figure can be found at: https://doi.org/10.48579/PRO/3LQH1M.
(TIFF)

S7 Fig Trait variation within the phenotypic spaces of F_2_s (blue) and the 254 G_0_ accessions (red) of *Arabidopsis thaliana*, based on 10 hypervolume calculations. Variation for the first three PCA dimensions of trait hypervolumes is presented. Hypervolume centroids, observed data points, and uniformly distributed random points generated by the algorithm used for hypervolume computation [27] are depicted by large circles, opaque dots, and semitransparent small dots, respectively. Hypervolumes with size equivalent to the mean of the 100 hypervolumes generated by resampling per group (F_2_s *versus* accessions) are shown. LA, leaf area; LDMC, leaf dry matter content; LNC, leaf nitrogen content; SLA, specific leaf area. The data underlying this figure can be found at: https://doi.org/10.48579/PRO/3LQH1M.
(ZIP)

S8 Fig Hypervolume sizes for F_2_s (blue) *versus* wild accessions (red) when including in the analyses imputed phenotypes from 104 G_0_ accessions lacking direct measurements. Size is represented in units of standard deviation to power five (the number of trait dimensions used for hypervolume computation). The result shown corresponds to one of the 10 hypervolume calculations. Bars denote SE. The data underlying this figure can be found at: https://doi.org/10.48579/PRO/3LQH1M and http://1001genomes.org/.
(TIFF)

S9 Fig Trait variation within the phenotypic spaces of F_2_s (blue) and wild accessions (red) of *Arabidopsis thaliana*, based on 10 hypervolume calculations including imputed phenotypes from 104 G_0_ accessions lacking direct measurements. Variation for the first three PCA dimensions of trait hypervolumes is presented. Hypervolume centroids, observed data points, and uniformly distributed random points generated by the algorithm used for hypervolume computation [27] are depicted by large circles, opaque dots, and semitransparent small dots, respectively. Hypervolumes with size equivalent to the mean of the 100 hypervolumes generated by resampling per group (F_2_s *versus* accessions) are shown. LA, leaf area; LDMC, leaf dry matter content; LNC, leaf nitrogen content; SLA, specific leaf area. The data underlying this figure can be found at: https://doi.org/10.48579/PRO/3LQH1M and http://1001genomes.org/.
(ZIP)

S10 Fig Manhattan plots showing SNPs associated with each analyzed trait in the 254 G_0_ accessions. The red line represents the significance threshold at *P* ≤ 0.05 with Bonferroni correction. LA, leaf area; LDMC, leaf dry matter content; LNC, leaf nitrogen content; SLA, specific leaf area. The data underlying this figure can be found at: https://doi.org/10.48579/PRO/3LQH1M and http://1001genomes.org/.
(ZIP)

S11 Fig Boxplots comparing environmental variables associated with phenotypically unique and common accessions. Phenotypically unique accessions are depicted in red, and phenotypically common accessions, in gray. Only environmental variables that were selected through the feature selection (Boruta) analysis are presented. Vpd, vapor pressure deficit; tas, mean daily air temperature; sand, soil sand content. Kruskal–Wallis test: **P < 0.01, ***P < 0.001. The data underlying this figure can be found at: https://doi.org/10.48579/PRO/3LQH1M, https://www.chelsa-climate.org/datasets/chelsa_climatologies/, and https://files.isric.org/soilgrids/latest/data_aggregated/1000m.
(TIFF)

S12 Fig Boxplots comparing the number of relict haplotypes between groups of genotypes. The number of relict haplotypes was evaluated across Spanish phenotypically unique (SP), Iberic phenotypically common and non-relict (IBER), Spanish relict (REL), and French (FR) accessions. The number of accessions (*N*) in each group is highlighted. Note log_10_ scale. Letters indicate pairwise significant differences from nonparametric Dunn test. Data from Lee and colleagues [21].
(TIFF)

S13 Fig Boxplots comparing vegetative, phenological, and reproductive traits between groups of genotypes. Eleven traits were evaluated across Swedish phenotypically unique (SW), Spanish phenotypically unique (SP), Spanish relict (REL), and French (FR) accessions: five vegetative (**a**), one phenological (**b**), and five reproductive (**c**) traits. The number of accessions (*N*) in each group for each comparison is highlighted. FT, flowering time; LA, leaf area; LDMC, leaf dry matter content; LNC, leaf nitrogen content; SLA, specific leaf area. Note log_10_ scale for FT and SLA, and square root scale for LNC and plant biomass. Nonparametric Dunn test: *P ≤ 0.05, **P < 0.01, ***P < 0.001. Dots denote marginal significance, and “ns” denotes non-significance in a Kruskal–Wallis test. The data underlying this figure can be found at: https://doi.org/10.48579/PRO/3LQH1M.
(ZIP)

S14 Fig Correlation circle of a dual multiple factor analysis (DMFA) with a “F_2_s (blue) *vs.* wild accessions (red)” contrast as grouping variable. This analysis was performed using the R package *FactorMineR* [80] on six traits. FT, flowering time; LA, leaf area; LDMC, leaf dry matter content; LNC, leaf nitrogen content; SLA, specific leaf area. The data underlying this figure can be found at: https://doi.org/10.48579/PRO/3LQH1M.
(TIFF)

## Abbreviations

BSLMM: Bayesian Sparse Linear Mixed Model
CEFE: Centre d’Ecologie Fonctionnelle et Evolutive
GWA: Genome-Wide Association
LA: leaf area
LDMC: leaf dry matter content
LES: leaf economics spectrum
LNC: leaf nitrogen content
MAF: minor allele frequency
PCA: principal component analysis
SLA: specific leaf area
VPD: vapor pressure deficit
