# Supplementary material for: Divergent and stabilizing selection shape the phenotypic space of Arabidopsis thaliana
Source: PLoS Biol. 2025 Dec 1;23(12):e3003536. doi: 10.1371/journal.pbio.3003536 (PMC12680341; doi:10.1371/journal.pbio.3003536)
Supplement: S1 Table — Information about their genetic group and country of origin, extracted from the 1,001 Genomes Project dataset (http://1001genomes.org/), is provided. (DOCX) [file pbio.3003536.s001.docx]

**S1 Table. List of the phenotypically unique accessions.** Information about their genetic group and country of origin, extracted from the 1,001 Genomes Project dataset (<http://1001genomes.org/>), is provided.

| **1001 Genomes ID** | **Genetic group** | **Country** |
| --- | --- | --- |
| 1006 | south_sweden | Sweden |
| 5867 | south_sweden | Sweden |
| 6030 | north_sweden | Sweden |
| 6071 | north_sweden | Sweden |
| 6074 | south_sweden | Sweden |
| 6077 | south_sweden | Sweden |
| 6097 | south_sweden | Sweden |
| 6099 | south_sweden | Sweden |
| 6101 | south_sweden | Sweden |
| 6102 | south_sweden | Sweden |
| 6111 | south_sweden | Sweden |
| 6119 | south_sweden | Sweden |
| 6126 | south_sweden | Sweden |
| 6128 | south_sweden | Sweden |
| 6131 | south_sweden | Sweden |
| 6145 | south_sweden | Sweden |
| 6194 | south_sweden | Sweden |
| 6216 | north_sweden | Sweden |
| 6258 | admixed | Sweden |
| 6413 | south_sweden | Sweden |
| 8242 | south_sweden | Sweden |
| 9386 | north_sweden | Sweden |
| 9409 | south_sweden | Sweden |
| 9416 | south_sweden | Sweden |
| 9452 | south_sweden | Sweden |
| 9470 | south_sweden | Sweden |
| 9481 | south_sweden | Sweden |
| 9548 | admixed | Spain |
| 9551 | admixed | Spain |
| 9577 | spain | Spain |
| 9593 | spain | Spain |
| 9594 | spain | Spain |
| 9824 | admixed | Spain |
| 9851 | western_europe | Spain |
| 9877 | admixed | Spain |
| 9885 | spain | Spain |
