# Supplementary material for: Divergent and stabilizing selection shape the phenotypic space of Arabidopsis thaliana
Source: PLoS Biol. 2025 Dec 1;23(12):e3003536. doi: 10.1371/journal.pbio.3003536 (PMC12680341; doi:10.1371/journal.pbio.3003536)
Supplement: S2 Table — R2 denotes the coefficient of determination. LA, leaf area; LDMC, leaf dry matter content; LNC, leaf nitrogen content; SLA, specific leaf area. (DOCX) [file pbio.3003536.s002.docx]

**S2 Table. Accuracy of trait imputation for the 104 G_0_ accessions for which phenotypes had not been measured directly.** R^2^ denotes the coefficient of determination. LA, leaf area; LDMC, leaf dry matter content; LNC, leaf nitrogen content; SLA, specific leaf area.

| **Trait** | **R²** | ***P* value** |
| --- | --- | --- |
| SLA | 0.26 | < 0.001 |
| LDMC | 0.25 | < 0.001 |
| LNC | 0.42 | < 0.001 |
| LA | 0.10 | < 0.001 |
| Plant biomass | 0.41 | < 0.001 |
| Flowering time | 0.49 | < 0.001 |
