# Supplementary material for: Divergent and stabilizing selection shape the phenotypic space of Arabidopsis thaliana
Source: PLoS Biol. 2025 Dec 1;23(12):e3003536. doi: 10.1371/journal.pbio.3003536 (PMC12680341; doi:10.1371/journal.pbio.3003536)
Supplement: S3 Table — LA, leaf area; LDMC, leaf dry matter content; LNC, leaf nitrogen content; SLA, specific leaf area. (DOCX) [file pbio.3003536.s003.docx]

**S3 Table. “Chip heritability” (‘PVE’) and the proportion of SNPs presenting a larger effect (‘pi’) on traits of wild accessions**. LA, leaf area; LDMC, leaf dry matter content; LNC, leaf nitrogen content; SLA, specific leaf area.

| **Trait** | **PVE (%)** | **pi (%)** |
| --- | --- | --- |
| SLA | 65.43 | 0.0119 |
| LDMC | 59.60 | 0.0130 |
| LNC | 91.13 | 0.0138 |
| LA | 29.95 | 0.0106 |
| Plant biomass | 82.49 | 0.0088 |
| Flowering time | 84.42 | 0.0067 |
