# Supplementary figures and images for: Divergent and stabilizing selection shape the phenotypic space of Arabidopsis thaliana
Source: PLoS Biol. 2025 Dec 1;23(12):e3003536. doi: 10.1371/journal.pbio.3003536 (PMC12680341; doi:10.1371/journal.pbio.3003536)

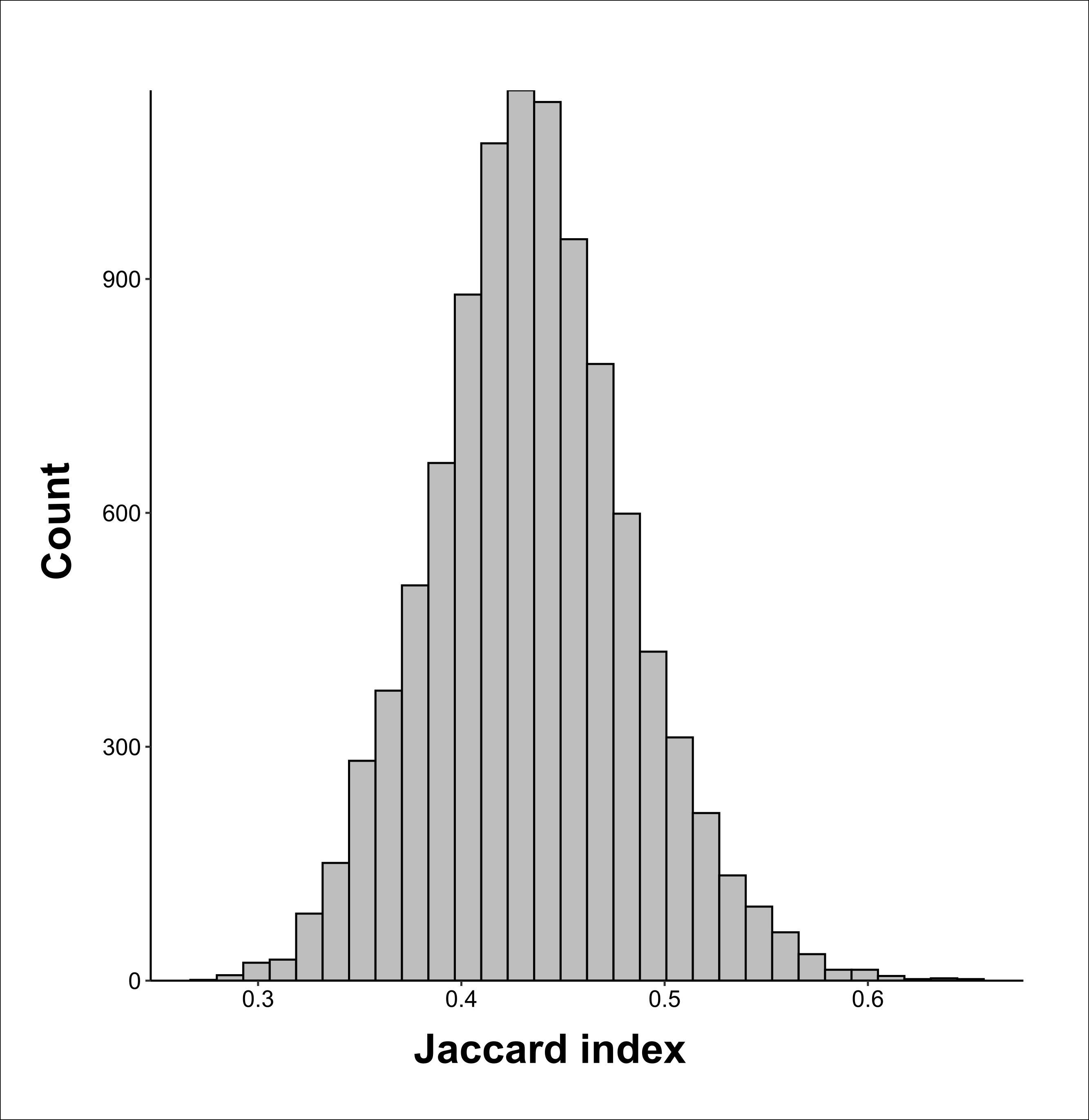

Supplement: S1 Fig — The distribution is based on 10,000 comparisons between resampled hypervolumes for each group. The result shown corresponds to one of the 10 hypervolume calculations. The data underlying this figure can be found at: https://doi.org/10.48579/PRO/3LQH1M. (TIFF) [file pbio.3003536.s004.tiff]

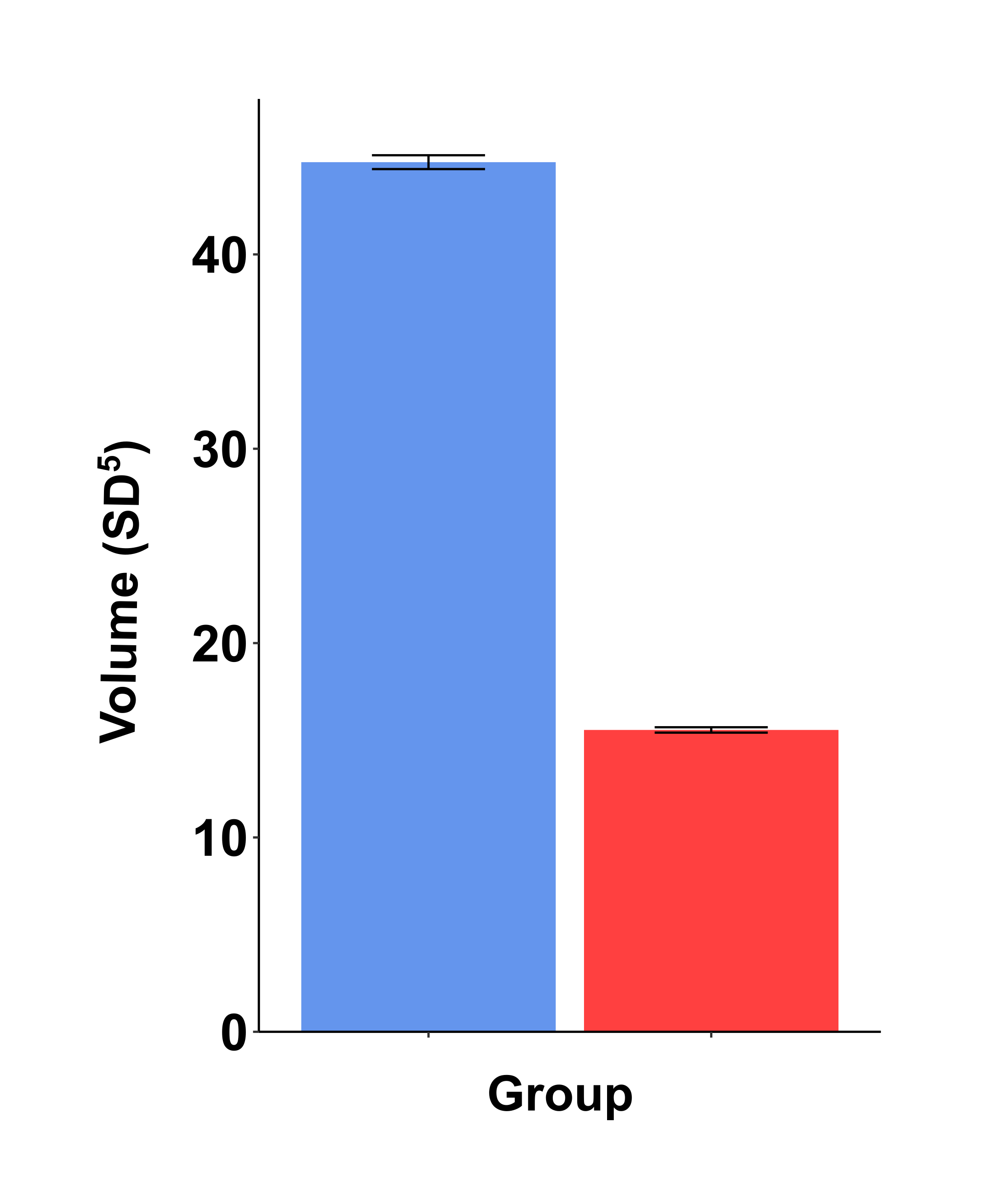

Supplement: S3 Fig — Size is represented in units of standard deviation to power five (the number of trait dimensions used for hypervolume computation). The result shown corresponds to one of the 10 hypervolume calculations. Bars denote SE. The data underlying this figure can be found at: https://doi.org/10.48579/PRO/3LQH1M. (TIFF) [file pbio.3003536.s006.tiff]

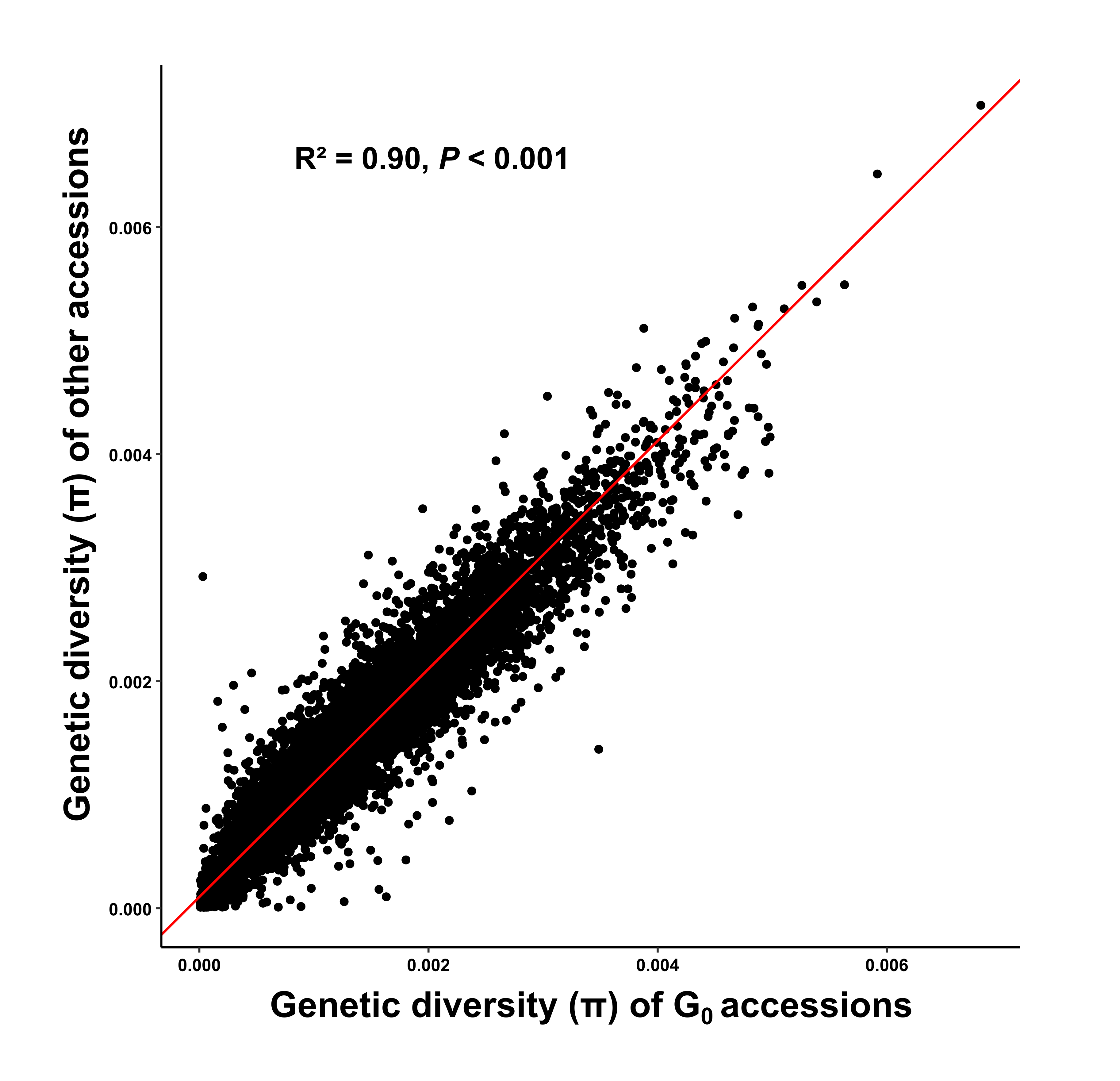

Supplement: S5 Fig — Each point represents one genomic region. The line was fitted with Standardized Major Axis (SMA) regressions, and R2 denotes the coefficient of determination. The data underlying this figure can be found at: https://doi.org/10.48579/PRO/3LQH1M and http://1001genomes.org/. (TIFF) [file pbio.3003536.s008.tiff]

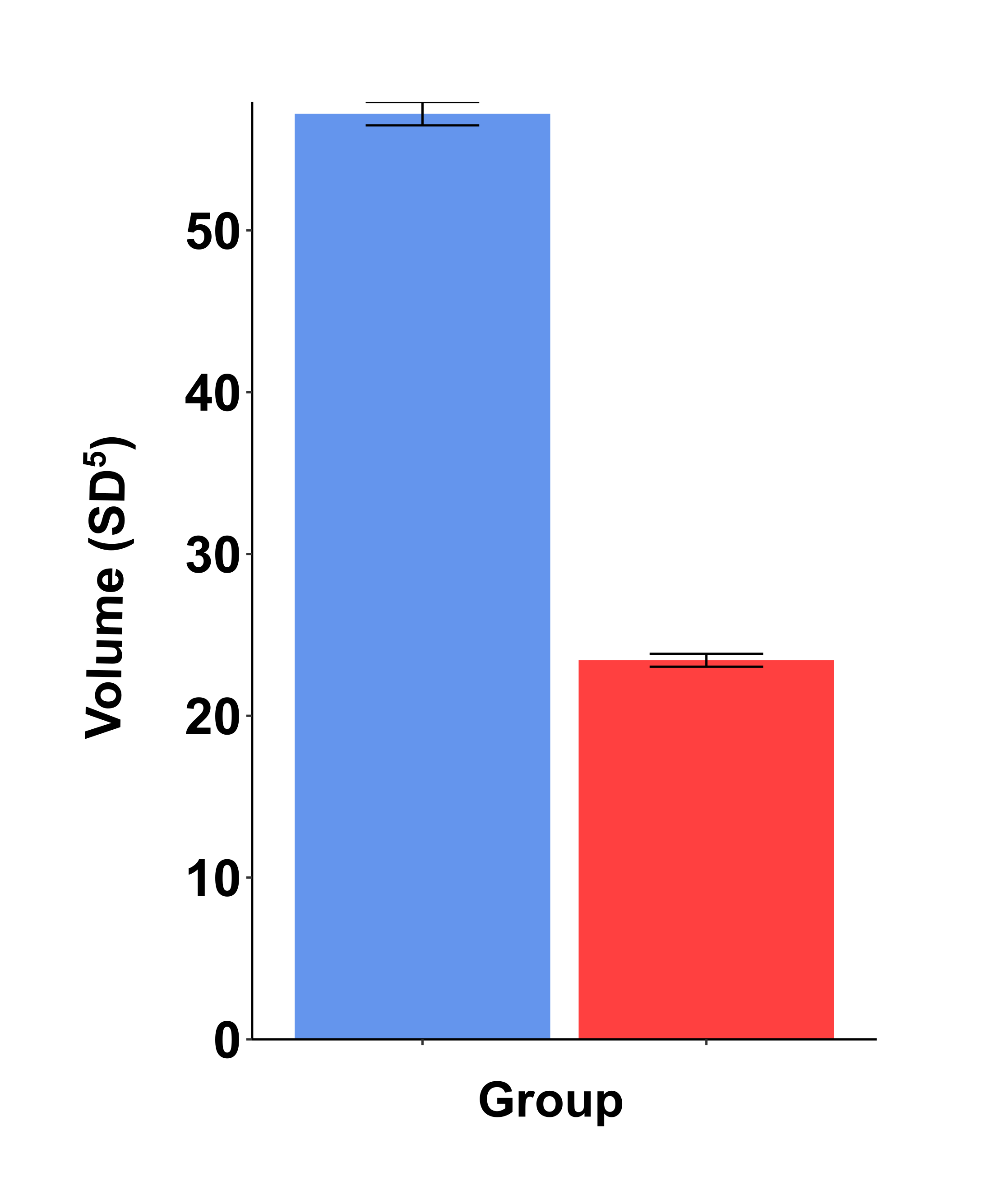

Supplement: S6 Fig — Size is represented in units of standard deviation to power five (the number of trait dimensions used for hypervolume computation). The result shown corresponds to one of the 10 hypervolume calculations. Bars denote SE. The data underlying this figure can be found at: https://doi.org/10.48579/PRO/3LQH1M. (TIFF) [file pbio.3003536.s009.tiff]

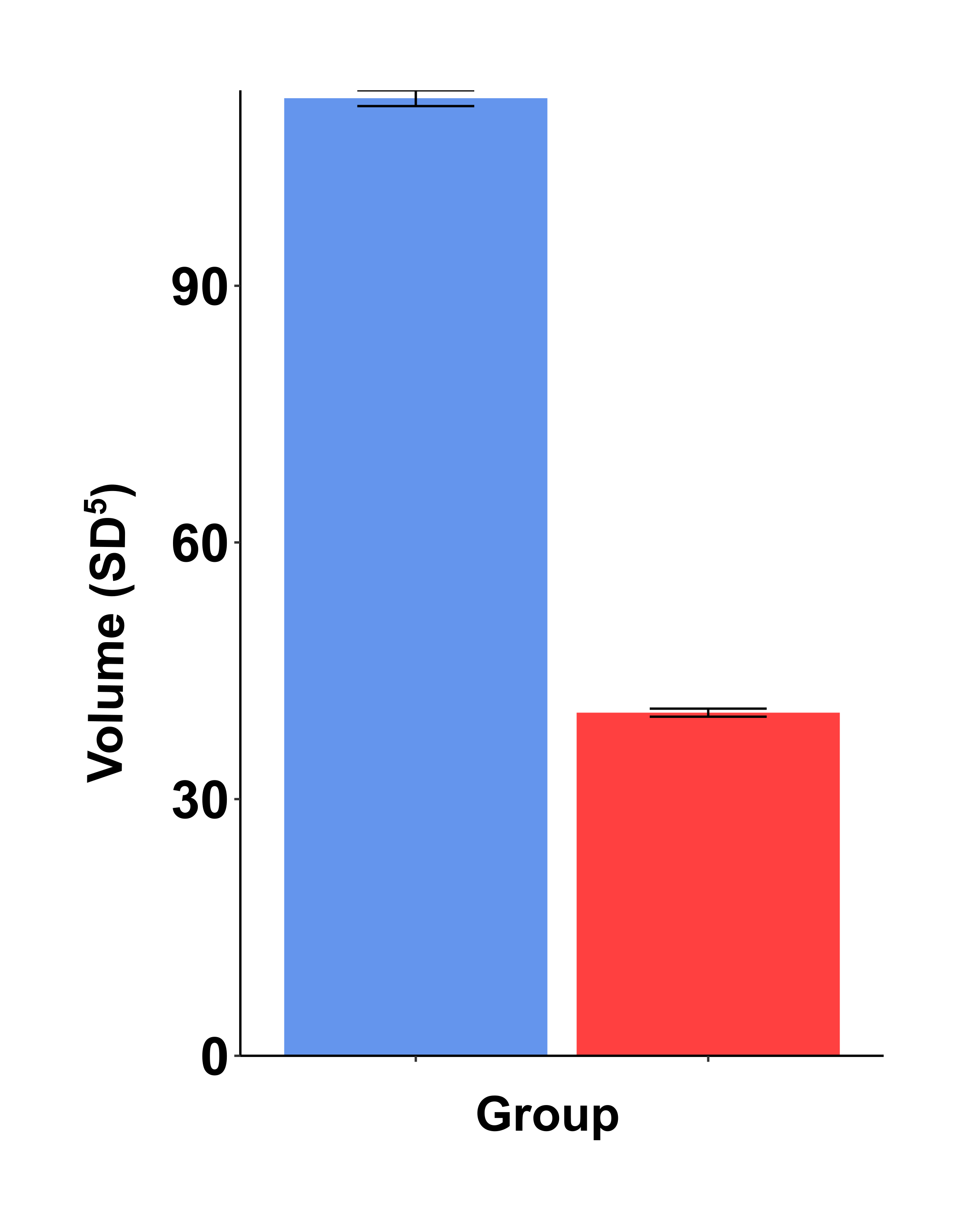

Supplement: S8 Fig — Size is represented in units of standard deviation to power five (the number of trait dimensions used for hypervolume computation). The result shown corresponds to one of the 10 hypervolume calculations. Bars denote SE. The data underlying this figure can be found at: https://doi.org/10.48579/PRO/3LQH1M and http://1001genomes.org/. (TIFF) [file pbio.3003536.s011.tiff]

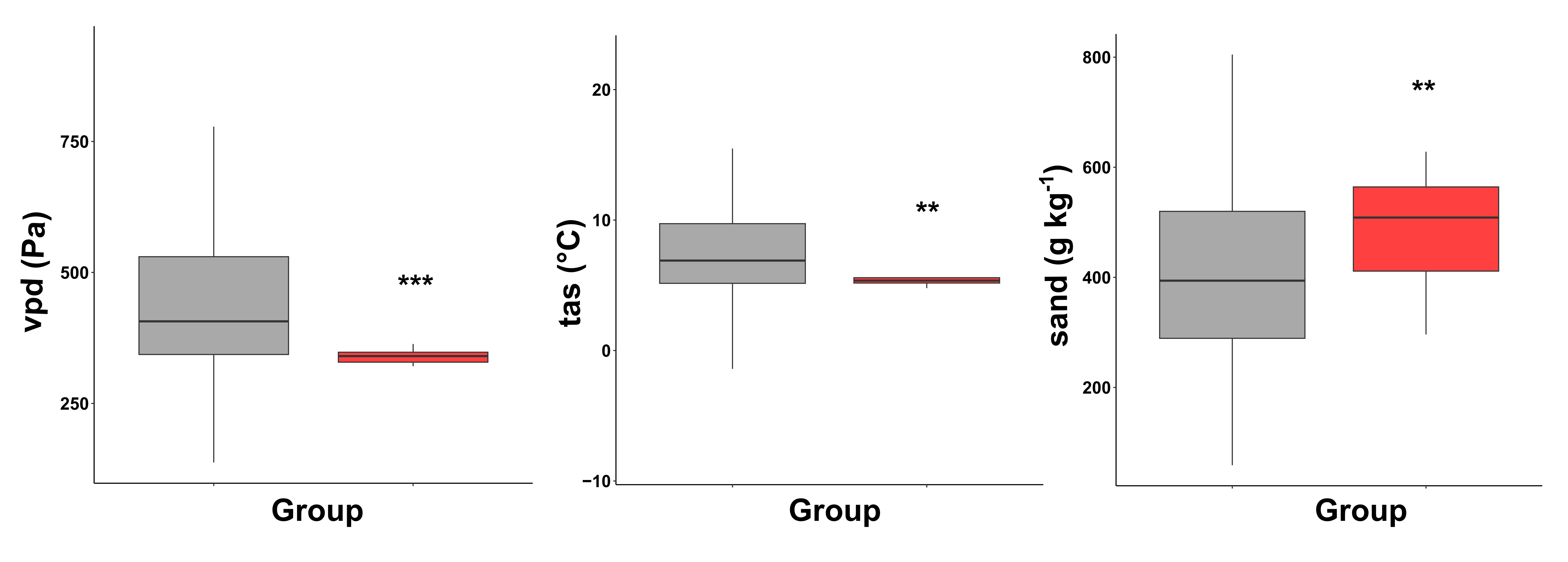

Supplement: S11 Fig — Phenotypically unique accessions are depicted in red, and phenotypically common accessions, in gray. Only environmental variables that were selected through the feature selection (Boruta) analysis are presented. Vpd, vapor pressure deficit; tas, mean daily air temperature; sand, soil sand content. Kruskal–Wallis test: **P < 0.01, ***P < 0.001. The data underlying this figure can be found at: https://doi.org/10.48579/PRO/3LQH1M, https://www.chelsa-climate.org/datasets/chelsa_climatologies/, and https://files.isric.org/soilgrids/latest/data_aggregated/1000m. (TIFF) [file pbio.3003536.s014.tiff]

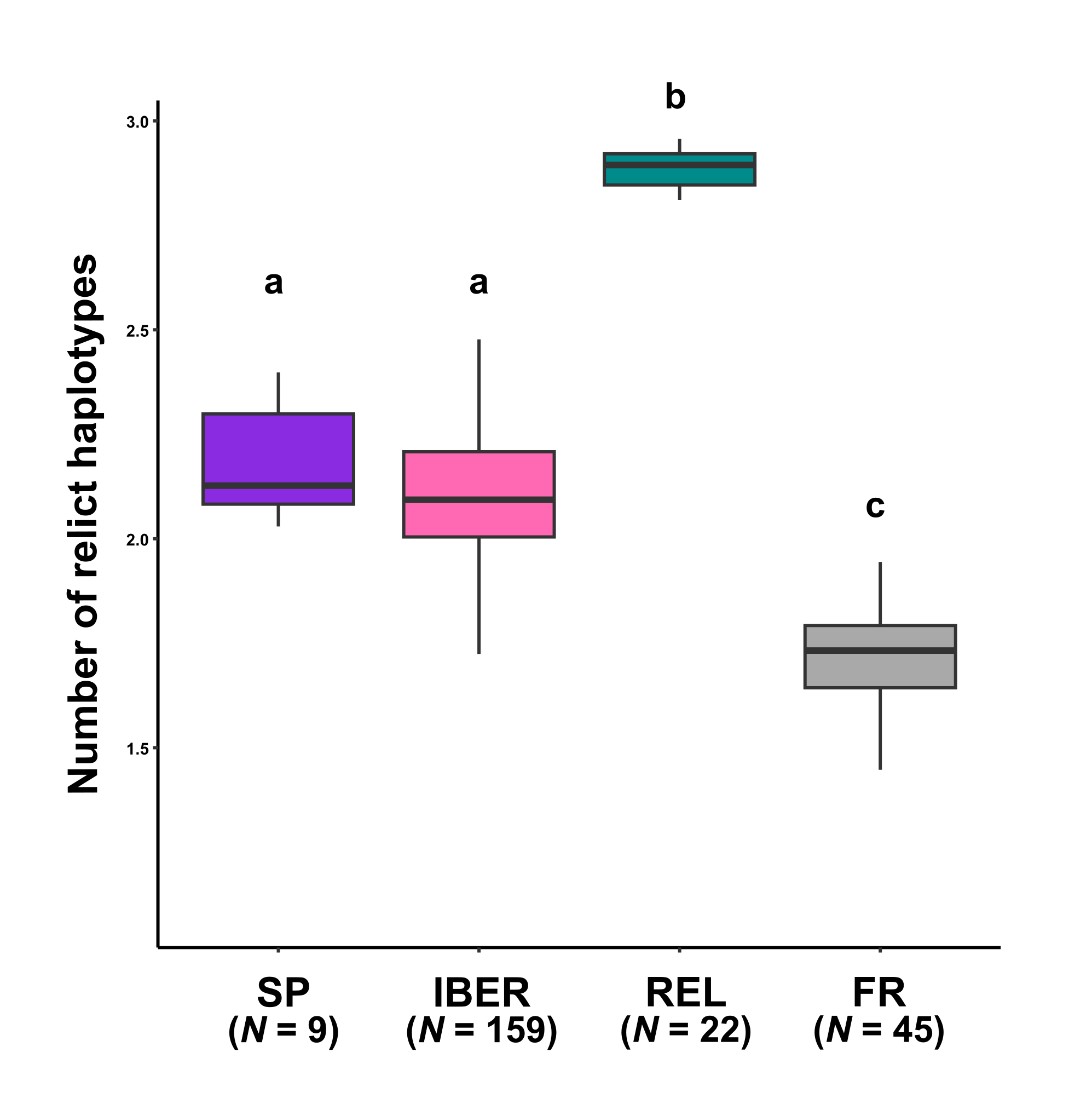

Supplement: S12 Fig — The number of relict haplotypes was evaluated across Spanish phenotypically unique (SP), Iberic phenotypically common and non-relict (IBER), Spanish relict (REL), and French (FR) accessions. The number of accessions (N) in each group is highlighted. Note log10 scale. Letters indicate pairwise significant differences from nonparametric Dunn test. Data from Lee and colleagues [21]. (TIFF) [file pbio.3003536.s015.tiff]

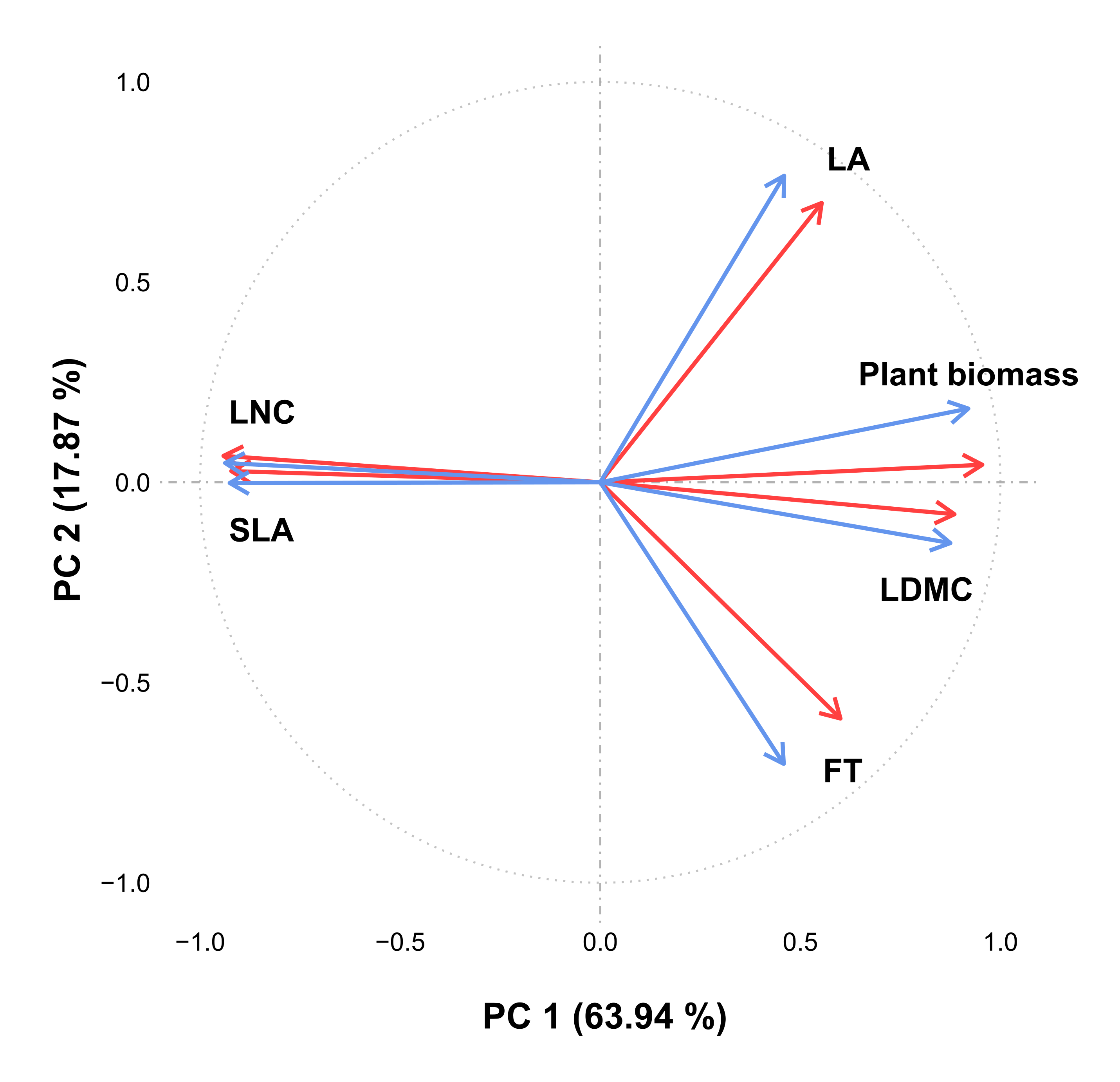

Supplement: S14 Fig — This analysis was performed using the R package FactorMineR [80] on six traits. FT, flowering time; LA, leaf area; LDMC, leaf dry matter content; LNC, leaf nitrogen content; SLA, specific leaf area. The data underlying this figure can be found at: https://doi.org/10.48579/PRO/3LQH1M. (TIFF) [file pbio.3003536.s017.tiff]
